# Heart transplantation as salvage therapy for progressive prosthetic valve endocarditis due to methicillin-resistant *Staphylococcus epidermidis* (MRSE)

**DOI:** 10.1186/s13019-016-0505-0

**Published:** 2016-07-11

**Authors:** J. P. Borde, G. Sitaru, W. H. Kopp, A. Ruhparwar, P. Ehlermann, F. Lasitschka, A. Dalpke, A. Heininger

**Affiliations:** Department of Internal Medicine, Ortenauklinikum Achern-Oberkirch, Division of Infectious Diseases, Josef-Wurzler-Straße 7, 77855 Achern, Germany; MVZ Clotten, Labor Dr. Haas, Dr. Raif & Kollegen, Merzhausener Straße 112a, 79100 Freiburg i.Br, Germany; Eurotransplant International Foundation, P.O. Box 2304, 2301 CH Leiden, The Netherlands; Department of Cardiac Surgery, Heidelberg University Hospital Center, Im Neuenheimer Feld 324, 69120 Heidelberg, Germany; Department of Cardiology, Heidelberg University Hospital Center, Im Neuenheimer Feld 410, 69120 Heidelberg, Germany; Institute of Pathology, Heidelberg University Hospital Center, Im Neuenheimer Feld 220/221, 69120 Heidelberg, Germany; Department of Infectious Diseases, Medical Microbiology and Hygiene, Heidelberg University Hospital Center, Im Neuenheimer Feld 324, 69120 Heidelberg, Germany; Department of Medicine, Division of Infectious Disease and Travel Medicine, Freiburg University Medical Center, Hugstetter Str. 55, 79106 Freiburg i.Br, Germany

**Keywords:** Methicillin-resistant *Staphylococcus epidermidis* (MRSE), Endocarditis, Rifampicin resistance, Prosthetic heart valve endocarditis, Heart transplantation

## Abstract

**Background:**

Prosthetic valve endocarditis (PVE) has the highest in-hospital mortality among all cases of infective endocarditis (IE), it is estimated at about 40 %. Orthotopic heart transplantation (OHT) as a measure of last resort, may be considered in selected cases where repeated surgical procedures and conservative efforts have failed to eradicate persistent or recurrent IE. Only few clinical data are available regarding this rare indication for OHT, since active IE has traditionally been considered as a contraindication for OHT.

**Case presentation:**

We report on a 55 year old male patient who underwent prosthetic valve replacement with a mechanical valved conduit ten years ago and developed now persistent PVE with severe complications due to methicillin-resistant *Staphylococcus epidermidis* (MRSE). Repeated surgical procedures and conservative efforts have failed to eradicate the pathogen. Regarding the lack of curative options, salvage OHT was discussed as a measure of last resort. 28 months after the first diagnosis of PVE, the patient was successfully transplanted and is now doing well under close follow-up (6 months post-OHT).

**Conclusions:**

PVE remains a challenging condition regarding diagnosis and treatment. The presented case underscores the urgent need for an integrated and multidisciplinary approach to patients with suspected and definitive IE - especially in PVE. OHT might be a feasible measure of last resort in selected patients with IE. Our case report adds published clinical experience to this rarely performed procedure and consolidates previous findings.

**Electronic supplementary material:**

The online version of this article (doi:10.1186/s13019-016-0505-0) contains supplementary material, which is available to authorized users.

## Background

Infective endocarditis (IE) is a severe condition with a high morbidity and mortality. Epidemiological studies report an annual incidence between 3 and 7 cases per 100.000 person years [1, 2]. In-hospital mortality ranges from 15 to 22 %, and overall 5-year mortality is approximately 40 % [3–5]. The male to female ratio is more than 2:1 [3]. Over the last years, *Staphylococcus aureus* has become the most frequently isolated pathogen in the context of IE [6–8]. Furthermore, there is an increasing number of prosthetic valve endocarditis patients and cardiac device related infective endocarditis cases in the last decades. National and international guidelines have been published regarding evidence-based diagnosis and treatment recommendations for native valve endocarditis (NVE), prosthetic valve endocarditis (PVE) and for cardiac device related infective endocarditis (CDRIE) [1, 9, 10]. The treatment of IE should nowadays be subject to an integrated multidisciplinary team approach, including cardiologists, infectious disease physicians, cardiac surgeons and microbiologists. In different clinical cohort studies [7, 11], up to 50 % of all IE cases required cardiac surgery during the active phase of the disease due to progressive valve regurgitations, perivalvular infection, vegetation size, recurrent embolisation and heart failure. The 2015 European Society of Cardiology (ESC) guideline mentioned cardiac transplantation as a measure of last resort, which “may be considered in extreme cases where repeated operative procedures have failed to eradicate persistent or recurrent PVE” [9]. However, only little experience and few clinical data are available regarding this indication for cardiac transplantation, since active IE has traditionally been regarded as a contraindication for orthotopic heart transplantation (OHT). Structured literature search using the search terms “endocarditis”and “heart transplantation”or “salvage treatment”retrieved only 18 patient cases, including a case series from France reporting 6 patients (and 10 previously published cases), who underwent OHT as salvage treatment for IE [12] (see Additional file 1: Table S1.). In the “Eurotransplant Zone”, there were 10 patients transplanted between 1995 and 2015 for the diagnosis IE. Median time to transplant was 62 days (range 1 – 180 days) and the median age of the transplant recipients was 48 years (range 33 – 62 years), 8/10 recipients were male and 8/10 were transplanted in an urgent/prioritized setting [personal communication]. OHT is a measure of last resort in IE. However, in the era of extreme donor organ shortage, further studies and published clinical experience are needed to evaluate very carefully, which endocarditis patients might benefit from OHT.

## Case presentation

We report on a 55 year old male patient with a history of severe aortic valve stenosis, ectasia of the aorta ascendens (48 mm) and reduced left ventricular function. He underwent prosthetic valve replacement with a mechanical valved conduit (St. Jude Medical Conduit CAVG 29 mm) in 2003. Anticoagulation was started with phenprocoumon. Implantation of a cardiac pacemaker (Medtronic Kappa KDR 503) due to atrioventricular block III° was necessary five days after initial heart surgery.

The patient presented to the emergency department in 03/2013 with a new sleep-onset weakness of the left arm and difficulties to speak. Emergency cranial computed-tomography (CCT) revealed ischemic brain lesions in the territory of the right middle cerebral artery. Neither atrial fibrillation nor valvular lesions or carotid artery stenoses were detected, when the patient was evaluated for causative mechanisms. Rehabilitation was finally completed with minimal residual neurological symptoms. Five months later, the patient was readmitted with fatigue, febrile temperatures, headache, nausea and progressive left-sided weakness and dysarthria. CCT scans after readmission showed signs of the preceding ischemic stroke in the territory of the right middle cerebral artery and new signs of progressive embolisation in the territory of the left middle cerebral artery with haemorrhage. C-reactive protein (CRP) was now 59 mg/l (ref. <5 mg/l) and the erythrocyte sedimentation rate (ESR) was 45 mm/h (ref. 0–30 mm/h). Sequentially obtained blood culture samples (5 of 5 sets) were positive for *Staphylococcus epidermidis* resistant to oxacillin (MRSE) (see Table 1). In line with additional minor DUKE criteria, predisposition, febrile temperatures and septic emboli, the definitive diagnosis of infective prosthetic valve endocarditis (PVE) was made. Antiinfective treatment with vancomycin plus rifampicin was started and the patient was transferred to a tertiary university medical center providing immediate facilities for cardiosurgical and neurosurgical interventions. The initial bedside transthoracic echocardiography on admission revealed no signs of valvular masses suggestive for vegetations, a moderate mitral regurgitation was noted. Evaluation via transesophageal echocardiography 48 h later showed a filiforme (7x4mm) mass at the prosthetic aortic valve and a perivalvular dehiscence of the mechanical valved conduit. Fortunately, at this time no surgical intervention was required.

**Table 1:** Evolving *Staphylococcus epidermidis* resistance patterns during the course of disease

	07/2013	05/2014	04/2015
	[Bloodculture isolates]	[Bloodculture isolates]	[Bloodculture isolates]
Daptomycin	S	S	S
Vancomycin	S	S	S
Penicillin G	R	R	R
Flucloxacillin/Oxa	R	R	R
Erythromycin	R	S	R
Clarithromycin	S	S	R
Tetracycline	R	S	S
Ampicillin + Sulb	R	R	R
Amoxicillin + Clav	R	R	R
Imipenem	R	R	R
Cefuroxim	R	R	R
Gentamicin	S	S	S
Moxifloxacin	R	I	R
Levofloxacin	R	R	R
Ciprofloxacin	R	R	R
Cotrimoxazol	S	R	R
Tigecyclin	S	S	S
Fosfomycin	S	S	S
Linezolid	S	S	S
Fusidinsaeure	S	S	S
Rifampicin	S	R	R
Clindamycin	S	S	R

**Fig. 1:**
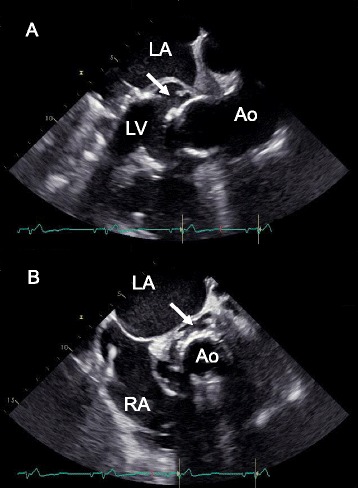
Transesophageal echocardiography (TEE) findings on cardiological follow-up evaluation 06/2014 after urgent prosthetic mitral valve replacement was successfully performed in 11/2013. In the different views, A and B, the abscess formation (*arrows*) around the mechanical valved conduit is shown, which has direct contact to the left ventricle (LV). LA denotes for the left atrium, RA right atrium and Ao aorta ascendens

In 11/2013 the patient developed progressive clinical signs of heart failure on the basis of severe mitral regurgitation, on transesophageal echocardiography destruction of the aortic-mitral continuity was noted, which made a mitral-valve repair intervention impossible. Urgent prosthetic valve replacement was successfully performed (St. Jude Medical MJ-501 33 mm). Histopathological samples showed signs of low grade chronic inflammation. Tissue samples from the surgical site and swab specimen from the left ventricular outflow tract (LVOT) were culturepositive for *S. epidermidis* (MRSE). In conclusion, new prosthetic valve material had to be inserted into a non-sterile situs despite antiinfective pretreatment and sterile bloodcultures. A curative approach seemed from that particular time on all but impossible and the patient was discharged with cotrimoxazol plus rifampicin as a timely indefinite oral suppressive treatment. Seven months after mitral valve replacement, in the context of a rising CRP and progressive fatigue, breakthrough bacteremia was detected. The MRSE strain isolated from sequential bloodcultures displayed now resistance against rifampicin (see Table 1). Bloodstream negativity was achieved after prolonged treatment with daptomycin plus clindamycin. From the infectious disease perspective, the presence of this now isolated “difficult to treat” MRSE strain, together with lacking surgical options to remove or revise prosthetic material resembled a palliative situation, since there were no substances for effective oral suppression therapy left. The main concern however, was that in the near future the progressive destruction of the periprosthetic tissue between the aortic and mitral valve (Fig. 1) might result in a fatal valve complication or progressive embolization to the brain. Regarding the lack of curative options, salvage OHT was discussed as a measure of last resort. During the pretransplant workup, prostate cancer (pT3a, pN0 (0/27), R1, L0, V0, M0; Gleason Score 3 + 4 = 7a) was diagnosed and the patient was delisted for OHT due to apparent malignancy. Radical prostatevesiculectomy was performed with adjuvant radiotherapy, after an interdisciplinary tumorboard decision. Twelve months later, under oral antiinfective suppressive therapy with moxifloxacin and clindamycin, the patient developed febrile temperatures with MRSE bacteremia – now displaying resistance against clindamycin and all fluoroquinolones. Fortunately, bloodstream negativity could be induced again with a prolonged course of daptomycin on an outpatient basis (OPAT). Finally, the patient was reassessed for OHT under stable conditions, bloodculture negativity and in line with oncological transplant requirements – 28 months after the first diagnosis of infective PVE, the patient was successfully transplanted. In the peri-transplantion period and for four weeks post-transplantion the patient received daptomycin plus fosfomycin. Bloodcultures remained sterile at any time and in particular after ending antiinfective treatment. He is now under close follow-up doing well (6 months post-OHT). Histopathological examination of the explanted organ and prosthetic material showed clear evidence of chronic active IE. Tissue specimen from aortic tissue were conclusive positive for *Staphylococcus epidermidis* on broadspectrum 16 s-PCR. From the isolated tissue samples the mecA gene encoding for oxacillin resistance was amplified.

## Discussion

Prosthetic valve endocarditis remains a challenging condition regarding diagnosis and treatment, despite improvements in nuclear imaging and molecular techniques. This distinct entity of IE has the highest in-hospital mortality among all IE cases, it is estimated at about 40 %. An observational before/after study from France, including 173 IE patients in the period before the team approach was implemented and 160 IE patients in the study period after a standardized protocol was established, reported a significant reduction in endocarditis related mortality and complications [13]. A very similar collaborative approach published in a study from Italy showed an improvement in NVE-related mortality [14]. Consequently, the “Endocarditis Team” was recommended as Ib in the 2014 AHA guideline for the management of patients with valvular disease and strongly supported (Class IIa recommendation) by the 2015 ESC endocarditis guideline [9]. Such teams should include specialists from various clinical disciplines - infectious disease specialists, cardiologists, cardiac surgeons, microbiologists, neurologists and neurosurgeons [9]. The presented case underlines the urgent need for an integrated and multidisciplinary approach to patients with suspected and definitive IE to prevent fatal complications and facilitate optimal treatment in the different clinical settings, ranging from primary to tertiary care providers. Retrospectively, the choice of antiinfective substances (for overview see Additional file 2: Table S2), combination therapy and aspects of dosing might be a matter of discussion – however, this would go beyond the scope of this case report. Overall, OHT as salvage therapy of IE remains a rare transplant indication. Only scarce clinical experience is available on the basis of several published case reports and one small case series [12]. It must be pointed out, that published data might be biased. Favourable outcomes are more likely to be submitted and published than treatment failure. Data retrieved from the Eurotransplant register indicate a high waiting list mortality with 10 patients that were transplanted and 8 patients died waiting for an organ of the total of 18 patients that were actually listed for OHT with the indication IE in the period between 1995 and 2015 [personal communication].

In the era of organ shortages, further clinical studies and structured follow-up data are needed to carefully evaluate patients with IE, who might be eligible for OHT. Repeated sets of sterile blood cultures, i.e., the absence or effective suppression of bacteremia, seems to be one important requirement for OHT in this particular clinical situation. With respect to OHT in the setting of bacteremia and PVE, there are no data available. Driveline infections (DLIs) in the context left ventricular assist devices (LVADs) and consecutive OHT might resemble very similar clinical situations [15]. A recently published small retrospective study, comparing patients transplanted with active DLI (*n* = 12) versus no DLI (*n* = 26) showed no differences in mortality and length of hospital stay [16]. However, the safety of OHT in patients with uncontrolled infections remains unclear.

## Conclusion

PVE remains a challenging condition regarding diagnosis and treatment. The presented case underscores the urgent need for an integrated and multidisciplinary approach to patients with suspected and definitive IE - especially in PVE. OHT might be a feasible measure of last resort in selected patients with IE. Our case report adds published clinical experience to this rarely performed procedure and consolidates previous findings.

## Abbreviations

AHA, American Heart Association; Ao, aorta; CCT, cranial computed tomography; CDRIE, cardiac device related infective endocarditis; DLI, drive line infection; ESC, European Society of Cardiology; IDSA, Infectious Disease Society of America; IE, infective endocarditis; LA, left atrium; LV, left ventricle; LVAD, left ventricular assist device; LVOT, left ventricular outflow tract; mecA, mecA gene encodes for methicillin resistance; MRSE, methicillin resistant staphylococcus epidermidis; NVE, native valve endocarditis; OHT, orthotopic heart transplantation; OPAT, outpatient antibiotic treatment; PVE, prosthetic valve endocarditis; RA, right atrium; RV, right ventricle

## Additional files

Additional file 1: Table S1. Prosthetic valve endocarditis – PVE; Native valve endocarditis – NVE; OHT – orthotopic heart transplantation (DOCX 15 kb) Additional file 2: Table S2. Course of antiinfective treatment. (DOCX 13 kb)
