# Supplementary material for: Heart transplantation as salvage therapy for progressive prosthetic valve endocarditis due to methicillin-resistant Staphylococcus epidermidis (MRSE)
Source: J Cardiothorac Surg. 2016 Jul 11;11:100. doi: 10.1186/s13019-016-0505-0 (PMC4939521; doi:10.1186/s13019-016-0505-0)
Supplement: Additional file 1: Table S1. — Prosthetic valve endocarditis – PVE; Native valve endocarditis – NVE; OHT – orthotopic heart transplantation (DOCX 15 kb) [file 13019_2016_505_MOESM1_ESM.docx]

| Patient No. | Sex, Age | Pathogen  (culture positivity or positive NAAT) | Cardiac lesions and cardiac comorbidities | Duration of reported follow-up and outcome after HT | Reference |
| --- | --- | --- | --- | --- | --- |
| 1 | Male, 58 | *Staphylococcus epidermidis* [MRSE] | PVE - aortic and mitral valve endocarditis, conduit endocarditis. Congestive heart failure. | 6 months, survived | presented case |
| 2 | Male, 48 | *Tropheryma whipplei* | NVE - severly reduced left ventricular function, aortic valve vegetation. Left-ventricular assist device as brigde to OHT. | 12 months, survived | Borne et al., 2015 |
| 3 | Female, 40 | *Gemella haemolysans* | PVE - congenital aortic coarctation, bicuspid aortic valve, bioprosthetic aortic valve replacement and root enlargement. | 7 months, survived | Ramchandani et al., 2014 |
| 4 | Male, 24 | *Staphylococcus aureus* [MSSA] | NVE - aortic valve endocarditis. Histopathology: acute necrotic myocarditis (multiple abscesses in the left ventricle) | 9 years, survived | Aymami et al., 2014 |
| 5 | Male, 55 | *Streptococcus pneumoniae* | NVE - aortic and tricuspid valve endocarditis. Histopathology: multiple cardiac abscesses. | 3 years, survived |  |
| 6 | Male, 53 | *Streptococcus agalactiae* | NVE - aortic valve endocarditis. Histopathology: Large cardiac abscess in the left ventricle. | 25 months, survived |  |
| 7 | Male, 35 | *Streptococcus pneumoniae* | NVE - aortic valve endocarditis. | 24 months, survived |  |
| 8 | Male, 37 | *Staphylococcus aureus* [MSSA] | PVE - prosthetic aortic valve endocarditis | 12 months, survived |  |
| 9 | Male, 64 | Not documented | PVE - prosthetic aortic and mitral valve endocarditis | 5 months, survived |  |
| 10 | Male, 28 | *Staphylococcus aureus* [MRSA] | NVE - aortic valve endocarditis | 12 months, survived | Pozzi et al., 2013 |
| 11 | Male, 58 | *Propionibacterium acnes* | PVE - prosthetic mitral valve endocarditis, cardiac pacemaker. | 68 months, survived | Huang et al., 2012 |
| 12 | Male, 31 | *Brucella melitensis* | PVE - prosthetic aortic valve endocarditis | 18 years, survived | Durante-Mangoni et al., 2011 |
| 13 | Male, 17 | Culture negative | PVE - prosthetic aortic valve endocarditis, Marfan syndrome, required second HT after relapse 5 years later | 30 months after second HT, survived | Guerrero et al., 2011 |
| 14 | Female, 25 | *Mycoplasma hominis* | PVE - prosthetic aortic and mitral valve endocarditis | 14 months, survived | Li et al., 2000 |
| 15 | Female, 44 | *Staphylococcus epidermidis* [MRSE] | PVE - prosthetic valve endocarditis, intracardiac defibrillator vegetations; previous OHT for hypertrophic cardiomyopathy. | 12 weeks, survived | Galbraith et al., 1999 |
| 16 | Male, 30 | *Coxiella burnetii* | PVE - prosthetic mitral valve endocarditis | 15 months, survived | Pulpon et al., 1994 |
| 17 | Male, 54 | *Staphylococcus aureus* [MRSA] | PVE - prosthetic mitral valve endocarditis; previous OHT for end-stage ischemic cardiomyopathy. | 6 years, survived | Blanche et al., 1994 |
| 18 | Female, 58 | *Staphylococcus epidermidis* | PVE - prosthetic mitral valve endocarditis | 25 months, survived | Park et al., 1993 |
| 19 | Male, 33 | *Poylmicrobial, Streptococcus mitis, Coagulase negative staphylococcus spp.,* | PVE - prosthetic aortic valve endocarditis | 5 years, survived | DiSesa et al., 1990 |
