# Supplementary material for: Heart transplantation as salvage therapy for progressive prosthetic valve endocarditis due to methicillin-resistant Staphylococcus epidermidis (MRSE)
Source: J Cardiothorac Surg. 2016 Jul 11;11:100. doi: 10.1186/s13019-016-0505-0 (PMC4939521; doi:10.1186/s13019-016-0505-0)
Supplement: Additional file 2: Table S2. — Course of antiinfective treatment. (DOCX 13 kb) [file 13019_2016_505_MOESM2_ESM.docx]

| Date | Course of antiinfective treatment |
| --- | --- |
| 08/2013 | First diagnosis of PVE, 5/5 bloodculture sets positive for *Staphylococcus epidermidis* (MRSE). Antiinfective therapy with vancomycin i.v. plus rifampicin 450mg 2x/d p.o. for 6 weeks – effective therapeutic drug monitoring for vancomycin (trough concentration range 10-15 µg/ml). |
| 11/2013 | Severe mitral regurgitation and clinical signs of heart failure, indication for urgent prosthetic valve replacement – culturepositive surgical samples for MRSE from LVOT. Antiinfective therapy with vancomycin i.v. plus rifampicin 300mg 2x/d p.o. for 8 weeks – effective therapeutic drug monitoring for vancomycin (trough concentration range 10-15 µg/ml). |
| 01/2014 | Initiation of oral antiinfective/suppressive therapy with cotrimoxazol 960mg 1x/d plus rifampicin 300mg 2x/d p.o.. |
| 04/2014 | Breakthrough bacteremia with MRSE “difficult to treat pathogen” (resistant to rifampicin), – first diagnosis of prostate cancer, uro-/oncologic therapy. Initiation of daptomycin 850mg 1x/d i.v. plus clindamycin 600mg 4x/d p.o. until 08/2014. |
| 08/2014 | Initiation of oral antiinfective/suppressive therapy with moxifloxacin 400mg 1x/d p.o. plus clindamycin 600mg 4x/d p.o. |
| 04/2015 | Breakthrough bacteremia with MRSE (resistance to rifampicin, clindamycin and fluoroquinolones), initiation of daptomycin 500mg 1x/d i.v. on outpatient basis (OPAT) for 6 weeks until 06/2015. |
| 06/2015 | Initiation of oral antiinfective/suppressive therapy with doxycycline 200mg 1x/d p.o. . |
| 12/2015 | OHT – surgical samples remained sterile on culture, PCR positivity for MRSE. Six week antiinfective course of daptomycin 850mg 1x/d i.v. plus fosfomycin 5g 4x/d i.v. peri-/ post-transplant. |
